# The synthetic triterpenoids CDDO-TFEA and CDDO-Me, but not CDDO, promote nuclear exclusion of BACH1 impairing its activity

**DOI:** 10.1016/j.redox.2022.102291

**Published:** 2022-03-17

**Authors:** Laura Casares, Rita Moreno, Kevin X. Ali, Maureen Higgins, Sharadha Dayalan Naidu, Graham Neill, Lena Cassin, Anders E. Kiib, Esben B. Svenningsen, Alberto Minassi, Tadashi Honda, Thomas B. Poulsen, Clotilde Wiel, Volkan I. Sayin, Albena T. Dinkova-Kostova, David Olagnier, Laureano de la Vega

**Affiliations:** a Jacqui Wood Cancer Centre, Division of Cellular Medicine, School of Medicine, University of Dundee, UK; b Institute of Clinical Sciences, Department of Surgery, Sahlgrenska Center for Cancer Research, University of Gothenburg, Gothenburg, Sweden; c Wallenberg Centre for Molecular and Translational Medicine, University of Gothenburg, Gothenburg, Sweden; d Department of Biomedicine, Health, Aarhus University, 8000, Denmark; e Department of Chemistry, Aarhus University, Denmark; f Department of Drug Science, University of Piemonte Orientale, Novara, Italy; g Department of Chemistry and Institute of Chemical Biology & Drug Discovery, Stony Brook University, Stony Brook, NY, 11794-3400, USA

**Keywords:** BACH1, HMOX1, CDDO, NRF2

## Abstract

The transcription factor BACH1 is a potential therapeutic target for a variety of chronic conditions linked to oxidative stress and inflammation, as well as cancer metastasis. However, only a few BACH1 degraders/inhibitors have been described. BACH1 is a transcriptional repressor of heme oxygenase 1 (HMOX1), which is positively regulated by transcription factor NRF2 and is highly inducible by derivatives of the synthetic oleanane triterpenoid 2-cyano-3,12-dioxooleana-1,9(11)-dien-28-oic acid (CDDO). Most of the therapeutic activities of these compounds are due to their anti-inflammatory and antioxidant properties, which are widely attributed to their ability to activate NRF2. However, with such a broad range of action, these compounds have other molecular targets that have not been fully identified and could also be of importance for their therapeutic profile. Herein we identified BACH1 as a target of two CDDO-derivatives (CDDO-Me and CDDO-TFEA), but not of CDDO. While both CDDO and CDDO-derivatives activate NRF2 similarly, only CDDO-Me and CDDO-TFEA inhibit BACH1, which explains the much higher potency of these CDDO-derivatives as HMOX1 inducers compared with unmodified CDDO. Notably, we demonstrate that CDDO-Me and CDDO-TFEA inhibit BACH1 via a novel mechanism that reduces BACH1 nuclear levels while accumulating its cytoplasmic form. In an *in vitro* model, both CDDO-derivatives impaired lung cancer cell invasion in a BACH1-dependent and NRF2-independent manner, while CDDO was inactive. Altogether, our study identifies CDDO-Me and CDDO-TFEA as dual KEAP1/BACH1 inhibitors, providing a rationale for further therapeutic uses of these drugs.

## 1 Background

The synthetic oleanane triterpenoid 2-cyano-3,12-dioxooleana-1,9(11)-dien-28-oic acid (CDDO) and its derivatives, including CDDO-methyl ester (CDDO-Me, also known as Bardoxolone methyl) and CDDO-trifluoroethyl amide (CDDO-TFEA), are a class of multifunctional drugs with anti-inflammatory and antioxidant properties that have a wide range of therapeutic uses, from neuroprotection to anticancer, in a variety of preclinical models [[1], [2], [3], [4], [5]]. These compounds were first identified as inducers of heme oxygenase 1 (HMOX1), an inducible enzyme with potent antioxidant and anti-inflammatory properties, and later as potent activators of the transcription factor NRF2 [6]. Extensive structure-activity studies led to the development of the most potent NRF2 activators known to date, with some of them, such as CDDO-Me and CDDO-DFPA, currently in advanced clinical trials [7,8] and others, like CDDO-TFEA (which has increased ability to cross the blood-brain barrier) showing promising results in preclinical models [9,10]. NRF2 is largely controlled at the protein stability level, and its main regulator, KEAP1 (Kelch-like ECH-associated protein 1), is a substrate adaptor for the Cul3-based E3 ubiquitin ligase, and in normal conditions, KEAP1 targets NRF2 for proteasomal degradation, keeping the levels of NRF2 low in cells [11]. KEAP1 is also a sensor for electrophiles, such as CDDO and its derivatives, which chemically modify cysteines in KEAP1 [12,13] preventing it from targeting NRF2 for degradation, leading to a rapid nuclear accumulation of NRF2 and transcription of its target genes, including heme oxygenase 1 (HMOX1) [11].

In addition to NRF2, the transcription of *HMOX1* is also regulated by BACH1 (broad complex, tramtrack and bric à brac and cap'n’collar homology 1), a transcription factor that competes with NRF2 for binding to sequences called antioxidant response elements (AREs) within its promoter region. Unlike NRF2 which activates *HMOX1* transcription, BACH1 represses it [[14], [15], [16], [17]]. While KEAP1 inhibitors/NRF2 activators induce the expression of numerous cytoprotective genes, BACH1 inhibitors/degraders activate only a limited subset of these genes, although they are extremely potent at inducing *HMOX1*.

Despite their therapeutic potential for a variety of conditions, including Huntington's and Parkinson's disease [18,19], spinal cord injury [20,21], ischemia/reperfusion injury [22], pulmonary fibrosis [23], and cancer [[24], [25], [26], [27], [28], [29], [30], [31]], only a few BACH1 inhibitors/degraders have been identified so far. The most widely used BACH1 degrader is hemin, a heme derivative. Hemin binds to BACH1, promoting its nuclear export and subsequent cytoplasmic degradation [[32], [33], [34]]. Other described degraders/inhibitors are the natural phytochemical cannabidiol [35], the synthetic compound HPP-4382 [27], and its derivatives [24], although their mechanisms of action are not clear. Based on the differential effect of BACH1 versus KEAP1 inhibitors, we expect drugs with dual activity, targeting both transcription factors, to have broader and stronger anti-inflammatory and antioxidant properties with potentially greater therapeutic value than drugs targeting either protein individually. In that regard, we have reported a chemical derivative of cannabidiol with dual activity and protective effects in a cellular model of Huntington's disease [19] and a recent work characterised a novel dual BACH1/KEAP1 inhibitor with protective effects in a Parkinson's model [18].

CDDO-derivatives are more potent than CDDO at inducing HMOX1 [6,36] and have a better therapeutic profile, but the reason for this increased activity is unclear. In this work we demonstrate that the CDDO derivatives CDDO-Me and CDDO-TFEA, are potent BACH1 inhibitors, while CDDO is not. This dual KEAP1 and BACH1 inhibition explains their enhanced potency as *HMOX1* inducers and may also explain some of their superior therapeutic profile.

## 2 Materials and methods

### 2.1 Cell culture

Cells were grown in RPMI (HaCaT and HK2) or DMEM (H1299, A549) containing 10% FBS at 37 °C and 5% CO_2_. LX2 cells were maintained in high glucose DMEM media with 2 mM l-Glutamine, without sodium pyruvate and with 2% FBS EmbryoMax™ (Sigma-Aldrich, St. Louis, MO, USA). HaCaT cells have been validated by STR profiling. LX2 cells were obtained from SIGMA, and HK2, H1299 and A549 cells were obtained from ATCC. All cell lines were routinely tested for mycoplasma. CRISPR-edited cells were produced as previously described [19,35,37]. Control cells, referred as HaCaT wild type (HaCaT WT), are the pooled population of surviving cells transfected with an empty pLentiCRISPRv2 vector treated with puromycin. The generation of HaCaT BACH1-KO and double NRF2/BACH1-KO [19], and NRF2-GOF and NRF2-KO [35,38,39] cells has been previously described. In short, the endogenous *BACH1* or *NFE2L2* gene, were edited by transfecting cells with pLentiCRISPR-v2 (a gift from Dr Feng Zhang, Addgene plasmid #52961) containing single-guide (sg) RNAs directed against *BACH1* (CGATGTCACCATCTTTGTGG and GACTCTGAGACGGACACCGA) or the KEAP1-binding domain within the *NFE2L2* locus (TGGAGGCAAGATATAGATCT). CRISPR-mediated gene editing with the sgRNA against NRF2 produced either NRF2-knockout clones (NRF2-KO) or NRF2-Gain-of-function clones (NRF2-GOF). NRF2-GOF clones were those in which the Cas9-mediated cleavage was repaired in frame but introducing indels (often deletions) within the KEAP1-binding domain (thus the smaller size of the NRF2-GOF as compared with the WT, as previously observed [38]. The generation of A549 BACH1-KO cells were produced as described before [40]. Lentiviral backbone used was pLentiCRISPRv2-blast (#98293, Addgene) expressing a sgRNA targeting human *BACH1 (*CCACTCAAGAATCGTAGGCC).

### 2.2 Antibodies and reagents

Antibodies against Beta-ACTIN (C-4), BACH1 (F-9) and LAMIN B2 (C-20) were obtained from Santa Cruz Biotechnology (Dallas, Texas, USA). Anti-NRF2 (D1Z9C) was obtained from Cell Signalling Technology (Danvers, MA, USA) and anti-HMOX1 was purchased from Biovision (San Francisco, CA, USA). Antibody against ALPHA-TUBULIN was obtained from Sigma-Aldrich (St. Louis, MO, USA). HRP-conjugated secondary antibodies were obtained from Life Technologies (Carlsbad, California, USA). Dimethyl sulfoxide (DMSO) was from Sigma-Aldrich. R,S-sulforaphane (SFN) was purchased from LKT Laboratories (St. Paul, MN, USA). (±)-TBE-31 was synthesized as described [41,42]. CDDO and CDDO-derivatives were obtained from Cayman Chemicals (Ann Arbor, MI, USA). MG132 was obtained from Santa Cruz Biotechnology, Leptomycin B from Cayman Chemicals, MLN4924 and Selinexor (KPT-330) from Selleckchem (Houston, TX, USA) and Actinomycin D and Cycloheximide from Sigma.

### 2.3 Plasmids

BACH1-RFP, and BACH1- C435, C46, C492, C646A (Hemin resistant) -RFP were generated as follows. BACH1 WT or Hemin-resistant inserts were synthesized and cloned into Plenti-CMV-MCS-RFP-SV-puro. Plenti-CMV-MCS-RFP-SV-puro was a gift from Jonathan Garlick & Behzad Gerami-Naini (Addgene plasmid # 109377).

### 2.4 Quantitative real time PCR (rt-qPCR)

RNA from cells was extracted using GeneJET RNA Purification Kit (Thermo Fisher Scientific) and 500 ng of RNA per sample was reverse-transcribed to cDNA using Omniscript RT kit (Qiagen) supplemented with RNase inhibitor according to the manufacturer's instructions. Resulting cDNA was analysed using TaqMan Universal Master Mix II (Life Technologies, Carlsbad, CA, USA) as well as corresponding Taqman probes. Gene expression was determined using a QuantStudio 7 Flex qPCR machine by the comparative ΔΔCT method. All experiments were performed at least in triplicates and data were normalised to the housekeeping gene HPRT1. Taqman probes used: HPRT1 Hs02800695_m1; HMOX1 Hs01110250_m1; AKR1B10 Hs00252524_m1.

### 2.5 Cell lysis and western blot

Cells were washed and harvested in ice-cold phosphate-buffered saline (PBS). For whole cell extracts, cells were lysed in RIPA buffer supplemented with phosphate and protease inhibitors. Lysates were sonicated for 15 s at 20% amplitude and then cleared by centrifugation for 15 min at 4 °C. For subcellular fractionation, cells were resuspended in 400 μl of low-salt buffer A (10 mM Hepes/KOH pH7.9, 10 mM KCL, 0.1 mM EDTA, 0.1 mM EGTA, 1 mM β-mercaptoethanol) and after incubation for 10 min on ice, 10 μl of 10% NP-40 was added and cells were lysed by gently vortexing. The homogenate was centrifuged for 10 s at 13,200 rpm, the supernatant representing the cytoplasmic fraction was collected and the pellet containing the cell nuclei was washed 4 additional times in buffer A. The pellet containing the nuclear fraction was then resuspended in 100 μl high-salt buffer B (20 mM Hepes/KOH pH7.9, 400 mM NaCl, 1 mM EDTA, 1 mM EGTA, 1 mM β-mercaptoethanol). The lysates were sonicated and centrifuged at 4 °C for 15 min at 13,200 rpm. The supernatant representing the nuclear fraction was collected. Protein concentration was determined using the BCA assay (Thermo Fisher Scientific, Waltham, MA, USA). Lysates were mixed with SDS sample buffer and boiled for 7 min at 95 °C. Equal amounts of protein were separated by SDS-PAGE, followed by semidry blotting to a polyvinylidene difluoride membrane (Thermo Fisher Scientific). After blocking of the membrane with 5% (w/v) non-fat dried milk dissolved in Tris buffered saline (TBS) with 0.1% v/v Tween-20 (TBST), membranes were incubated with the primary antibodies overnight at 4 °C. Appropriate secondary antibodies coupled to horseradish peroxidase were detected by enhanced chemiluminescence using ClarityTM Western ECL Blotting Substrate (Bio-Rad, Hercules, CA, USA). Resulting protein bands were quantified and normalised to each lane's loading control using the ImageStudio Lite software (LI-COR). For whole cell extracts, the protein of interest was normalised against ACTIN or GADPH. LAMIN was used as an internal control for nuclear extracts and TUBULIN or GADPH were used as controls for cytoplasmic extracts.

### 2.6 Cell viability assay

Alamar Blue (Thermo Fisher Scientific) was used to determine cell viability after drug treatment. HaCaT cells were seeded in 96-well plates to 50–60% confluency and treated the next day with the corresponding compounds for 48 h. After treatment, Alamar Blue was added to the wells (1:10 ratio) and after 4 h of incubation at 37 °C the fluorescence was measured (excitation 550 and an emission at 590 nm) using a microplate reader (Spectramax m2). Viability was calculated relative to the DMSO treated control.

### 2.7 Cell migration and invasion assays

Transwell invasion assays were performed with 6.5-mm inserts with 8.0-μm-pore membrane. Cells were treated for 6 h with the corresponding compounds, and then cells (7.5 × 10^4^/well) were resuspended in serum-free medium in the upper chamber with the corresponding compounds. The bottom chamber contained complete medium with 10% FBS supplemented with the corresponding compounds to avoid any concentration gradient of the compounds. The inserts were precoated with a 1:30 dilution of Matrigel (Corning 356234). After 16 h, cells in the upper chamber were removed with a humidified cotton swab, and invading cells on the other side of the membrane were fixed with PFA, stained with crystal violet, and photographed under a bright-field microscope (5X). The area covered by cells on each field of views was quantified with ImageJ on at least five fields per well.

### 2.8 Statistical analysis

Experiments were repeated at least 2–5 times with multiple technical replicates to be eligible for the indicated statistical analyses. Data were analysed using Graphpad Prism statistical package. All results are presented as mean ± SD unless otherwise mentioned. The differences between groups were analysed using one-way ANOVA.

## 3 Results

### 3.1 CDDO-Me and CDDO-TFEA, but not CDDO, reduce BACH1 levels

**Fig. 1:**
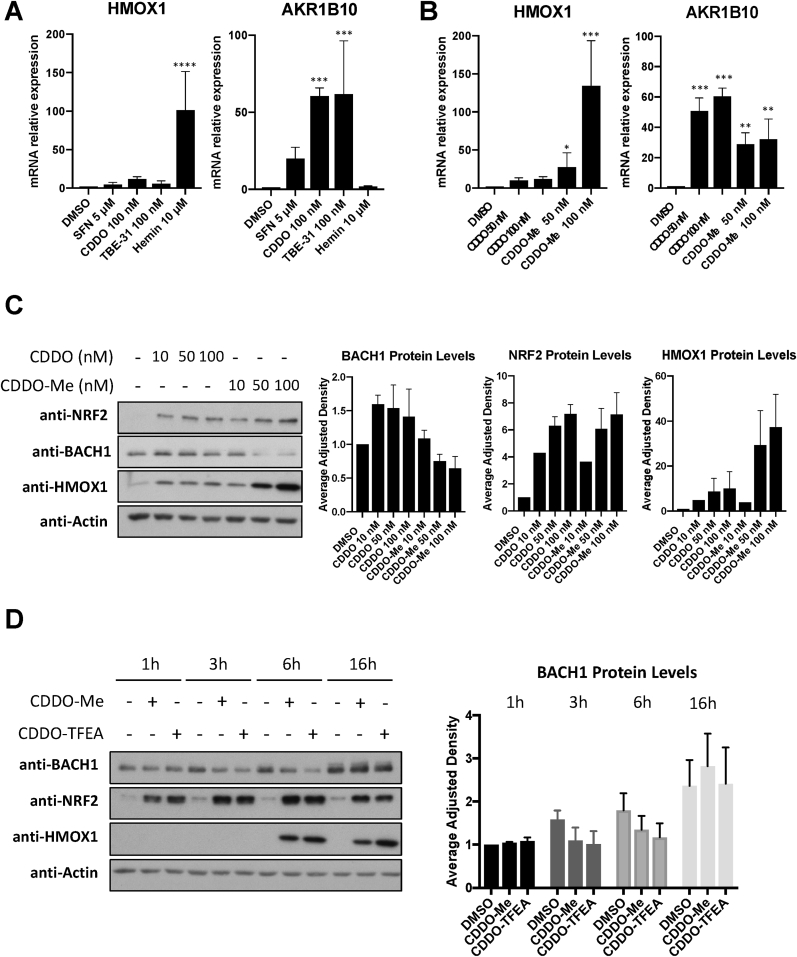
CDDO-Me and CDDO-TFEA, but not CDDO, reduce BACH1 levels. (A) HaCaT cells were treated with either DMSO (0.1%, v/v), SFN (5 μM), CDDO (100 nM), TBE-31 (100 nM) or Hemin (10 μM) for 16 h. Cells were lysed and mRNA levels of *HMOX1* and *AKR1B10* were analysed by qRT-PCR, using *HPRT1* as a housekeeping gene. ***P ≤ 0.001, ****P ≤ 0.0001. (B) As in A, but HaCaT cells were treated with either DMSO (0.1%, v/v) or increasing concentrations of CDDO or CDDO-Me. After 16 h cells were harvested and lysed and mRNA levels of *HMOX1* and *AKR1B10* were analysed by real-time qPCR. Data were normalised using *HPRT1* as an internal control (n = 3) and are expressed relative to the DMSO treated sample. *P ≤ 0.05, **P ≤ 0.01, ***P ≤ 0.001. (C) HaCaT cells were treated with DMSO (0.1%, v/v) or increasing concentrations of CDDO or CDDO-Me. Five hours later, cells were harvested and lysed. Protein levels of NRF2, BACH1, HMOX1 and ACTIN were analysed by Western Blot. Left panel shows a representative blot and right panels show quantification of NRF2 and HMOX1 protein levels against the loading control. Data represent means ± SD (n = 3) and are expressed relative to the DMSO-treated samples. (D) HaCaT cells were treated with either DMSO (0.1%, v/v), CDDO-Me (100 nM) or CDDO-TFEA (100 nM) for 1 h, 3 h, 6 h or 16 h. Cells were harvested, lysed and analysed for the levels of the indicated proteins. Left panel is a representative blot; right panels are the quantification of BACH1 levels (n = 3). Data are expressed relative to the DMSO-treated sample at time 1 h, which is set to 1.

We have previously shown in immortalised human keratinocytes (HaCaT cells), that the classical NRF2 activator sulforaphane (SFN) is a weak *HMOX1* inducer (but a very good inducer of the NRF2 transcriptional target *AKR1B10*), while BACH1 degraders such as hemin, strongly induce *HMOX1* (in an NRF2-independent manner) without affecting *AKR1B10* expression [19,35]. This emphasizes that although *HMOX1* has often been used as a surrogate for NRF2 activity, in some cases *AKR1B10* induction might be a more appropriate reporter for NRF2 activation while *HMOX1* induction is a better surrogate for BACH1 inhibition. To answer whether the observed limited effect of SFN on *HMOX1* in HaCaT cells is a general phenomenon for NRF2 activators, we compared three potent NRF2 activators (SFN, CDDO and TBE31) against a BACH1 degrader (hemin) for their ability to induce *HMOX1* in these cells. As shown in Fig. 1A, all three NRF2 activators were weak *HMOX1* inducers when compared with hemin but potent inducers of *AKR1B10* expression.

Since CDDO-Me is more potent than CDDO at inducing *HMOX1* expression in some cellular models, we tested whether CDDO-Me and CDDO had a differential effect on *HMOX1* transcription in HaCaT cells. CDDO-Me was significantly more potent (12 fold) than CDDO at inducing *HMOX1* expression, although both compounds were equally potent at inducing *AKR1B10* (Fig. 1B), suggesting that their differential effect on HMOX1 must be NRF2-independent. Next, we hypothesised that, in addition to activating NRF2, CDDO-Me might be targeting BACH1. To test this, we compared the effect that CDDO and CDDO-Me had on BACH1 and NRF2 protein levels. As shown in Fig. 1C, CDDO-Me, but not CDDO, moderately reduced BACH1 protein levels and greatly induced HMOX1, while both compounds equally stabilised NRF2. Since other CDDO-derivatives are also potent *HMOX1* inducers, we hypothesised that they might also reduce BACH1 protein levels. To test this, we compared the effect of various CDDO-derivatives on BACH1 and NRF2 protein levels as well as *HMOX1* and *AKR1B10* expression. Although still moderately, all tested derivatives were more potent than CDDO at reducing BACH1 levels (Suppl. Fig. S1A). Of those, CDDO-TFEA and CDDO-Me were the most potent at inducing *HMOX1* expression (Suppl. Fig. S1B). All compounds (CDDO and derivatives) induced *AKR1B10* to a similar extent (Suppl. Fig. S1B). Based on their potency, we focused on CDDO-TFEA and CDDO-Me (structures shown in Suppl. Fig. S1C) and performed a time course analysis of their effect on BACH1 levels. Our results show that BACH1 reduction appears to be maximal between three and 6 h, and that this effect is not observed at 16 h (Fig. 1D). Neither CDDO-TFEA nor CDDO-Me reduced cell viability at the concentrations used in various cellular systems (Suppl. Fig. S1D).

### 3.2 The differential effect of CDDO, CDDO-Me and CDDO-TFEA on *HMOX1* expression is due to BACH1 inhibition

**Fig. 2:**
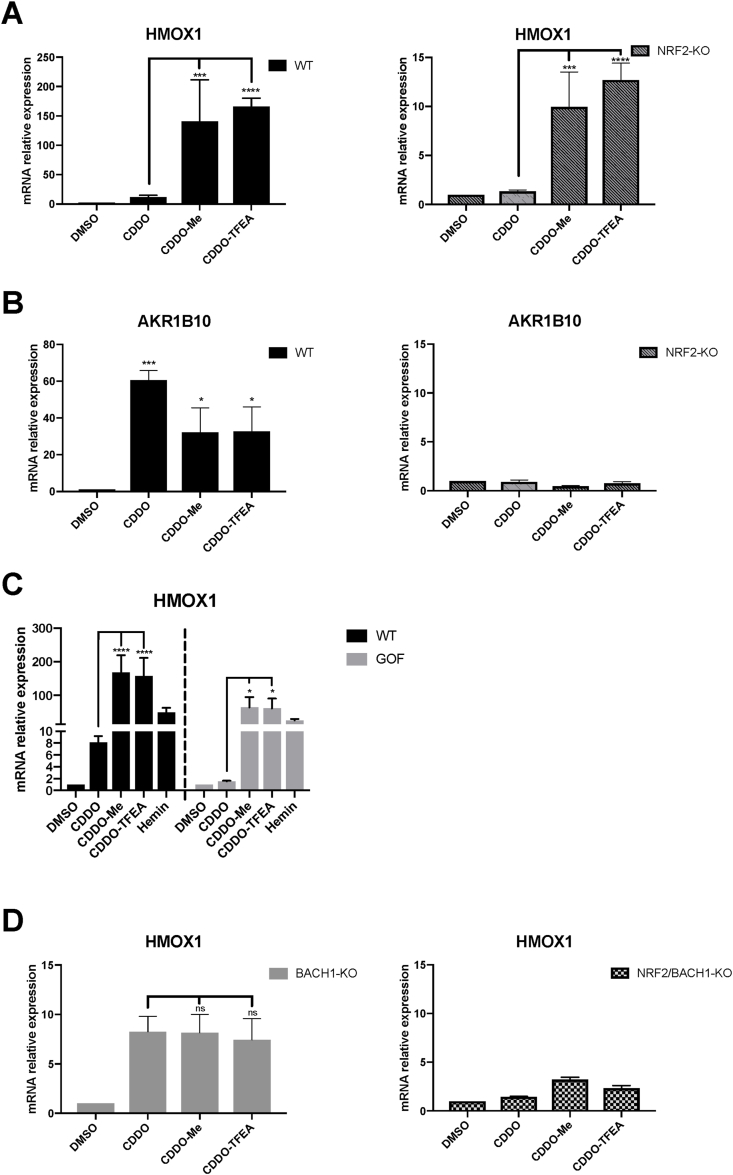
The differential effect of CDDO, CDDO-Me and CDDO-TFEA on *HMOX1* expression is due to BACH1 inhibition. (A,B) HaCaT WT or NRF2-KO cells were treated with either DMSO (0.1%, v/v), CDDO (100 nM), CDDO-Me (100 nM) or CDDO-TFEA (100 nM) for 16 h. Samples were collected and mRNA levels of *HMOX1* (A) and *AKR1B10* (B) were analysed via real-time qPCR, using *HPRT1* as an internal control. Data are expressed relative to the DMSO-treated samples in each cell line (DMSO in WT and NRF2-KO cells set to 1). *P ≤ 0.05, ***P ≤ 0.001, ****P ≤ 0.0001. (C) HK2 Control (WT) and NRF2-GOF cells were treated with DMSO, CDDO (100 nM), CDDO-Me (100 nM), CDDO-TFEA (100 nM) or Hemin (10 μM) for 16 h. *HMOX1* mRNA levels were analysed using RT-qPCR and *HPRT1* as a housekeeping gene. Data are expressed relative to the DMSO-treated samples in each cell line (DMSO in WT and NRF2-GOF cells set to 1). (D) HaCaT BACH1-KO and HaCaT NRF2/BACH1-KO cells were treated as in (A). Levels of *HMOX1* were analysed by qRT-PCR as previously described. *HMOX1* levels in the DMSO samples of each cell line were set to 1 and the rest of the data are expressed relative to their corresponding DMSO sample. *P ≤ 0.05, **P ≤ 0.01, ***P ≤ 0.001.

Reportedly, some CDDO-derivatives still increase HMOX1 protein levels in the absence of NRF2 [36], although the factor responsible for that induction has not been identified. To test whether in our system the differential effect of CDDO-TFEA and CDDO-Me versus CDDO was dependent on NRF2, we compared wild type (WT) and NRF2-KO HaCaT cells. We found that although in NRF2-KO cells *HMOX1* induction in response to the compounds was reduced (Suppl. Fig. S2A), both CDDO-TFEA and CDDO-Me were more potent than CDDO at inducing *HMOX1* in both WT cells (12–14 times more), and NRF2-KO cells (7–10 times more) (Fig. 2A), demonstrating that the differential effect between CDDO and CDDO-TFEA/Me was indeed not related to NRF2. On the other hand, *AKR1B10* induction in WT cells was similar for the three compounds and was completely abolished in the absence of NRF2 (Fig. 2B). We used a complementary approach with an immortalised human proximal tubular kidney cell line (HK2) to test if the NRF2-independent differential effect of CDDO-Me and CDDO-TFEA on *HMOX1* was cell-type specific. Using CRISPR/Cas9 gene editing, we produced an isogenic HK2 cell line with hyperactive NRF2 that cannot be further stabilised by activators (NRF2-GOF cells) (Cell line validation in Suppl. Fig. S2B). CDDO weakly induced *HMOX1* in the HK2-WT cells, while it failed to induce *HMOX1* any further in the NRF2-GOF cell line. In contrast, CDDO-Me and CDDO-TFEA were potent *HMOX1* inducers in both WT and NRF2-GOF cells (Fig. 2C), confirming that the differential HMOX1 induction mediated by CDDO versus CDDO-Me and CDDO-TFEA does not depend on NRF2 stabilisation. In agreement with the results obtained in HaCaT cells, the three compounds equally induced *AKR1B10* in WT HK2 cells but failed to induce it further in NRF2-GOF HK2 cells (Suppl. Fig. S2C).

As BACH1 is a key regulator of *HMOX1* expression, we hypothesised that the differential effect between CDDO and the two CDDO-derivatives must be due to their differential activity on BACH1, and that the strong effect of CDDO-Me and CDDO-TFEA on *HMOX1* expression relates to the combination of NRF2 stabilisation and BACH1 reduction. To test this, we compared the three compounds in BACH1-KO and in BACH1/NRF2-KO HaCaT cells. In BACH1-KO cells, the differential effect between CDDO and the two CDDO-derivatives on *HMOX1* was lost (Fig. 2D *left panel*), suggesting that BACH1 is indeed responsible for that effect (comparison between Fig. 2D and A), and that NRF2 (or another factor) might be responsible for the remaining observed induction. In fact, in double BACH1/NRF2-KO cells the effect of the compounds on *HMOX1* expression was largely abolished, highlighting the relevance of both factors regulating *HMOX1* (Fig. 2D *right panel*).

### 3.3 Both CDDO-Me and CDDO-TFEA reduce BACH1 nuclear levels while accumulating cytoplasmic BACH1 levels in a NRF2-independent manner

**Fig. 3:**
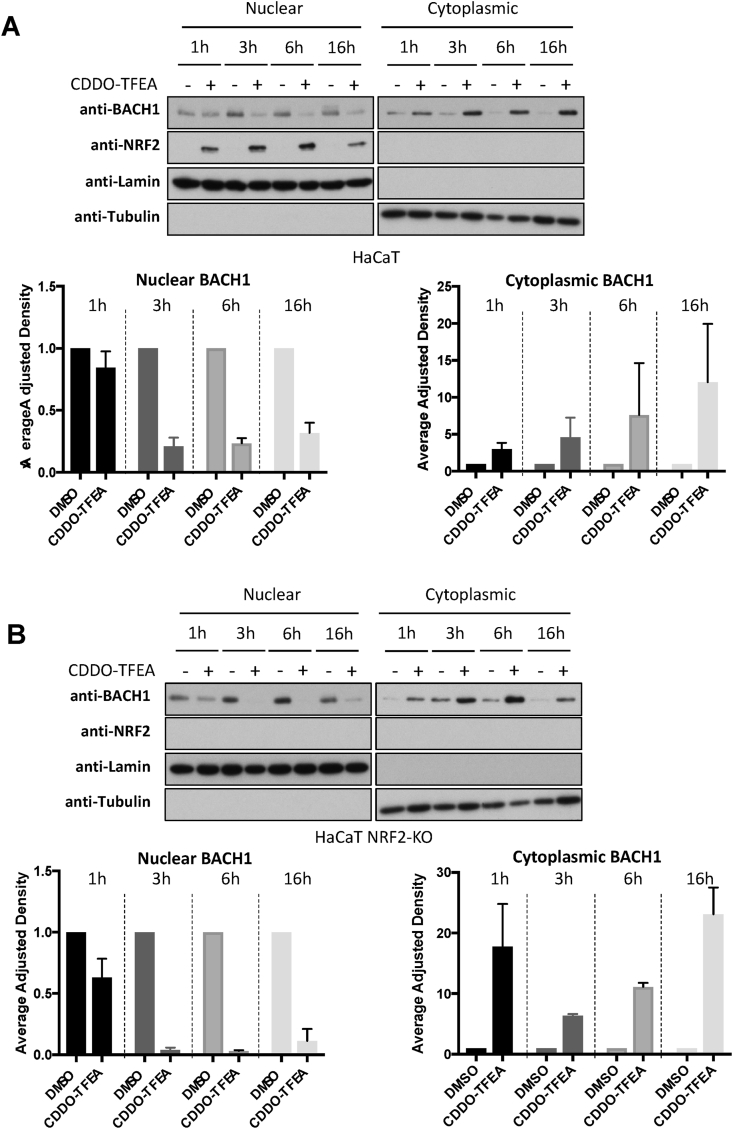
Both CDDO-Me and CDDO-TFEA reduce nuclear BACH1 while increasing cytoplasmic BACH1 levels. (A–B) HaCaT WT cells (A) and NRF2-KO cells (B) were treated with DMSO (0.1%, v/v) or CDDO-TFEA (100 nM) for 1 h, 3 h, 6 h or 16 h. Cells were harvested and nuclear and cytosolic fractions were isolated and analysed for the levels of the indicated proteins. Upper panel is a representative blot; lower panels are the quantification of BACH1 nuclear and cytoplasmic levels (n = 2). Data are expressed relative to the DMSO-treated samples for each time point (which were set to 1) and were normalised against their respective loading control. (G).

Our results demonstrate that CDDO-Me and CDDO-TFEA, but not CDDO, reduce the levels of BACH1, and that this reduction is responsible for their differential effect on *HMOX1* expression. However, the observed reduction of BACH1 levels was very moderate and less than expected based on the strong *HMOX1* induction obtained (which was similar to that obtained with the potent BACH1 degrader hemin, Fig. 1A and B). As some of the compounds that target BACH1 for degradation do so by first inducing its nuclear export [33], we wondered whether CDDO-Me and CDDO-TFEA might also affect the balance between nuclear/cytoplasmic BACH1 and whether the compounds could be differently affecting nuclear (the active pool) and cytoplasmic BACH1. To test this, we performed time course experiments in combination with subcellular fractionation. As shown in Fig. 3A, treatment with the two CDDO-derivatives led to a strong and sustained reduction of nuclear BACH1, while increasing its cytoplasmic abundance. This might explain the moderate effect observed on total BACH1 levels (as the cytoplasmic accumulation would mask its nuclear reduction) and the strong *HMOX1* induction (due to the strong BACH1 nuclear reduction as that is the transcriptionally active pool).

Additionally, as these compounds are potent NRF2 activators and NRF2 induces BACH1 expression [40,43], we tested whether NRF2 was necessary for the effect of CDDO-Me and CDDO-TFEA on BACH1 nuclear and cytoplasmic levels. To do this, we performed a similar time course experiments in NRF2-KO HaCaT cells. The absence of NRF2 did not affect the reduction in nuclear BACH1 nor its cytoplasmic accumulation (Fig. 3B), strongly suggesting that NRF2 is not required for either of these effects. In agreement, potent NRF2 activators such as CDDO or TBE31 did not induce BACH1 cytoplasmic accumulation or promote its nuclear reduction (Suppl Fig. S3A).

### 3.4 How are CDDO-Me and CDDO-TFEA affecting BACH1 levels?

The nuclear reduction and cytoplasmic accumulation of BACH1 in response to CDDO-TFEA/Me could be explained in different ways:

1 The two effects are not linked: e.g. CDDO-Me and CDDO-TFEA induce BACH1 nuclear degradation and independently BACH1 cytoplasmic accumulation, either by increasing the protein stability or the transcript levels of BACH1.
2 The two effects are linked: e.g. CDDO-Me and CDDO-TFEA affect the balance between nuclear and cytoplasmic BACH1 (i.e inducing BACH1 nuclear export or inhibiting nuclear import)

We next tested these two possible hypotheses:

### 3.5 Are CDDO-Me and CDDO-TFEA reducing BACH1 nuclear levels by increasing its degradation?

**Fig. 4:**
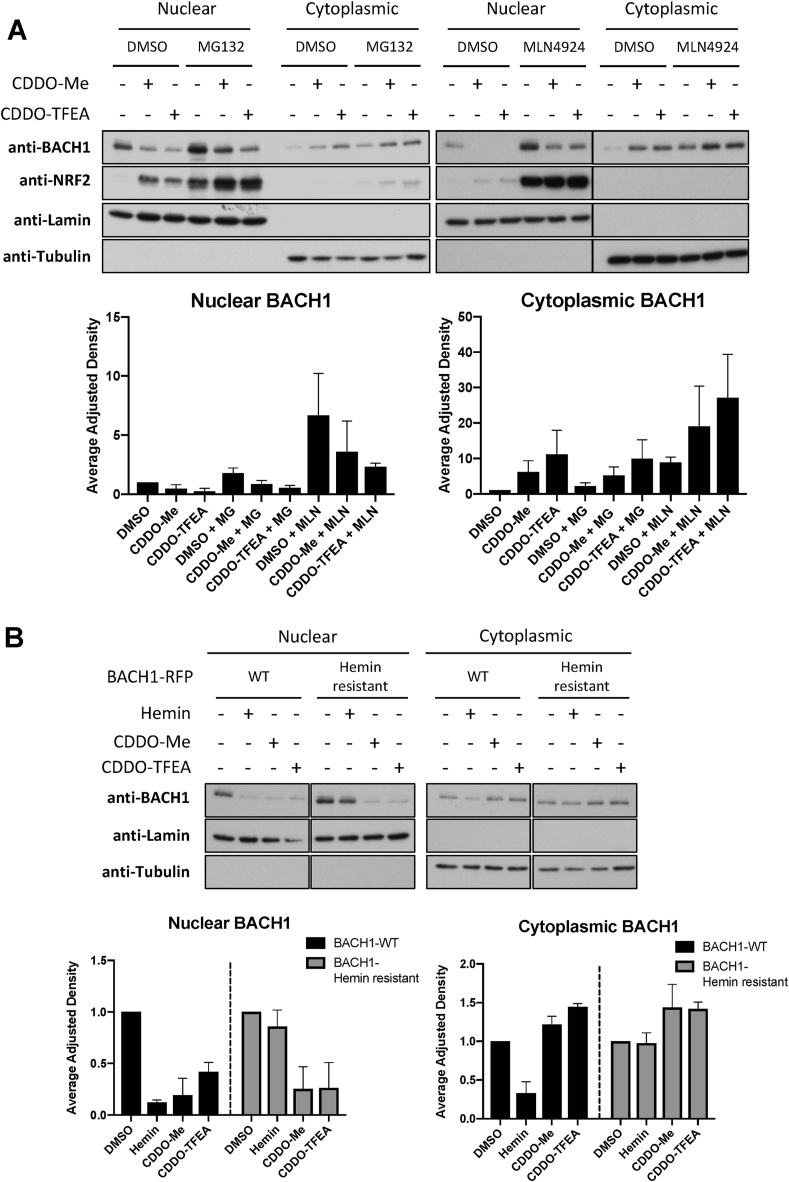
CDDO-Me and CDDO-TFEA affect BACH1 levels in a proteasome independent manner and have a mechanism of action different than hemin. (A) HaCaT cells were incubated with either DMSO (0.1%, v/v), MG132 (10 μM) or MLN4924 (2 μM) for 1 h. After that, either DMSO (−), CDDO-Me (100 nM) or CDDO-TFEA (100 nM) was added. Six hours later, cells were harvested and nuclear/cytoplasmic fractions were isolated and analysed for their levels of BACH1 and NRF2. Upper panel is a representative blot and lower panels are the quantifications of nuclear and cytoplasmic BACH1 levels normalised against their corresponding loading control. Data represent means ± SD (n = 3) and are expressed relative to the DMSO sample. (B) HaCaT BACH1-KO cells reconstituted with either BACH1-RFP-WT or BACH1-RFP-Hemin resistant mutant were treated with DMSO (−), Hemin (10 μM), CDDO-Me (100 nM) or CDDO-TFEA (100 nM) for 6 h. Cells were harvested and nuclear/cytoplasmic fractions were isolated and analysed for their levels of BACH1. Upper panel is a representative blot and lower panels are the quantifications of nuclear and cytoplasmic BACH1 levels normalised against their corresponding loading control. Data represent means ± SD (n = 3) and are expressed relative to their DMSO control (each cell line against their own DMSO, which was set as 1).

The two main pathways controlling protein degradation are the ubiquitin-proteasome system and autophagy. To study the involvement of the ubiquitin-proteasome system we used MG132 (proteasome inhibitor) and MLN 4924 (an inhibitor of NEDD8 activating enzyme, which acts by inhibiting all Cullin RING ligases). Although both inhibitors increased the basal levels of BACH1, neither of them abolished the effect of CDDO-TFEA/Me on BACH1 (Fig. 4A, Suppl. Fig. S4A), suggesting that degradation of BACH1 via the proteasome is not the main (or at least not the only) mechanism by which these two CDDO-derivatives reduce levels of BACH1. To address the potential role of autophagy, we used the autophagy inhibitor bafilomycin A1 (BAF-A1), which did not impair the effect of CDDO-Me/TFEA on BACH1 protein levels (Suppl Figs. S4B and S4C). Interestingly, BAF-A1 significantly increased the levels of BACH1, suggesting that basal levels of BACH1 are regulated by both proteasomal degradation and autophagy.

Hemin (the best-characterised BACH1 degrader) binds to BACH1, promoting its proteasomal degradation, and thus our results suggest that CDDO-Me, CDDO-TFEA and hemin might have different mechanisms of action. To address this, we reconstituted BACH1-KO cells with either BACH1-WT or a BACH1 hemin-resistant mutant, in which four cysteines in the heme-binding site were mutated to alanine (Hemin-resistant) [33,44]. Although both hemin and the two CDDO-derivatives efficiently reduced nuclear levels of BACH1-WT, only CDDO-TFEA/Me reduced the levels of the hemin-resistant BACH1 mutant (Fig. 4B). These results further confirm that the mechanism of BACH1 reduction by CDDO-TFEA/Me is different from that of hemin.

### 3.6 Are CDDO-Me and CDDO-TFEA regulating BACH1 by affecting its transcription?

We first tested if CDDOs affected BACH1 transcription. We measured the transcription levels of *BACH1* in response to CDDO and the two CDDO-derivatives at 2 different time points. As shown in Suppl. Fig. S4D, none of the compounds affected its transcription at the 3-h time point, while all of them (as expected for NRF2 activators) induced *BACH1* transcription in the long term (16 h). To test whether the accumulation of cytoplasmic BACH1 in response to CDDO-Me and CDDO-TFEA was a consequence of changes in its transcription, we used compounds to inhibit either protein synthesis (cycloheximide, a protein synthesis inhibitor) or transcription (actinomycin D, a DNA-directed RNA synthesis inhibitor). Neither of these inhibitors blocked BACH1 cytoplasmic accumulation (or its nuclear reduction) in response to CDDO-TFEA/Me (Suppl Fig. S4E), suggesting that synthesis of new proteins (and their transcription) is not needed for the effect of the two CDDO-derivatives on BACH1.

### 3.7 Are CDDO-Me and CDDO-TFEA regulating BACH1 nucleocytoplasmic transport?

Based on the moderate effect of CDDO-derivatives in total BACH1 and their strong effect reducing nuclear BACH1 while accumulating it in the cytoplasm, we hypothesise that the main mechanism involved must be related to BACH1 nucleocytoplasmic transport. Many nuclear export substrates contain a nuclear export signal (NES) that binds the export receptor CRM1. However, not all proteins that shuttle between the nucleus and cytoplasm use CRM1 to do so, and CRM1-independent nuclear export pathways have been identified [[45], [46], [47], [48], [49]]. To address whether the changes in nuclear and cytoplasmic BACH1 in response to CDDO-TFEA/Me are related to a CRM1-dependent nuclear export mechanism (as the most common mechanism reported for BACH1 regulation) we tested the effect of two CRM1 inhibitors (leptomycin B and selinexor) (Suppl. Figs. S4F and S4G). Although both inhibitors induced a basal accumulation of BACH1, neither of them abolished its nuclear reduction nor its cytoplasmic accumulation in response to CDDO-TFEA/Me. The inhibitors did not impair the moderate reduction of total BACH1 in response to CDDO-TFEA/Me either. These results suggest that CRM1 is not involved, but we cannot rule out the use of alternative nuclear export mechanisms or impairment of the nuclear import pathway.

### 3.8 CDDO-Me and CDDO-TFEA reduce lung cancer cell invasion in a BACH1-dependent and NRF2-independent manner

**Fig. 5:**
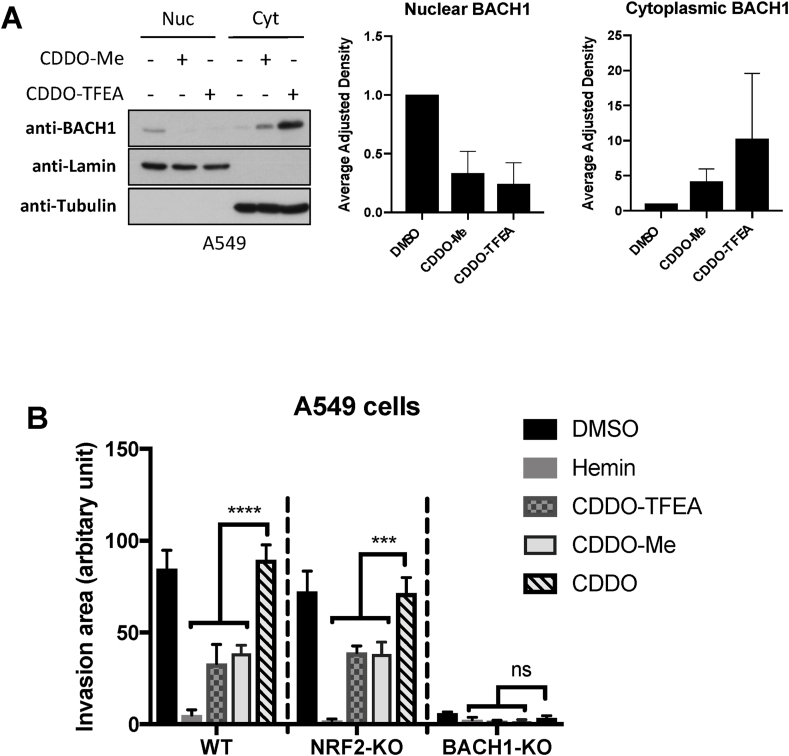
CDDO-Me and CDDO-TFEA reduce lung cancer cell invasion in a BACH1-dependent and NRF2-independent manner. (A) A549 cells were treated with DMSO (0.1%, v/v), CDDO-Me (100 nM) or CDDO-TFEA (100 nM). Six hours later cells were harvested and subcellular fractionation was performed. BACH1 protein levels were analysed via western blot. Panels on the left show a representative blot; panels on the right are the corresponding BACH1 nuclear and cytoplasmic quantifications, which were normalised against their internal control (i.e., LAMIN for nuclear and TUBULIN for cytoplasmic levels). Data represent means ± SD (n = 3) and are expressed relative to the DMSO-treated samples. (B) WT, NRF2-KO, or BACH1-KO A549 cells were treated with DMSO, CDDO (100 nM), CDDO-Me (100 nM), CDDO-TFEA (100 nM) or hemin (15 μM) for 6 h, followed by transwell invasion assays that were performed in presence of the inhibitors.

Due to the key role of BACH1 in different pathological scenarios such as chronic conditions and cancer, we tested whether CDDO-Me and CDDO-TFEA also reduced BACH1 levels in various relevant cell lines such as the immortalised human proximal tubular kidney cell line HK2, the human hepatic stellate cell line LX2 or the lung cancer cell lines H1299 and A549. Although both CDDO-derivatives were still robust inducers of *HMOX1* (and significantly more potent than CDDO) in all cell lines tested, they did not affect BACH1 total levels even at high concentration (Suppl. Figs. S5A–S5D). However, when we studied the nuclear and cytoplasmic BACH1 pool separately, we found that both CDDO-Me and CDDO-TFEA strongly reduced nuclear BACH1 while increasing its cytoplasmic levels (Fig. 5A and Suppl. S5E-G). These experiments show that the ability of CDDO-Me and CDDO-TFEA to reduce BACH1 nuclear levels is conserved in both untransformed and cancer cells, and further confirm that these compounds mainly affect the nuclear/cytoplasmic distribution of BACH1 and not its total levels.

BACH1 promotes lung cancer cell migration and invasion by inducing the expression of metabolic genes (such as HK2) leading to an increase in glycolysis [40], and thus BACH1 inhibitors have the potential to reduce the spread of lung cancer cells. To test the relevance of the BACH1 nuclear reduction mediated by CDDO-Me and CDDO-TFEA we used an *in vitro* model of lung cancer cell invasion. Due to the role of BACH1 promoting cancer cell invasion and the lack of redundancy with NRF2, we hypothesised that BACH1 inhibitors would reduce cancer cell invasion, while NRF2 activators would not. To study the NRF2-independent role of CDDOs we used the very invasive A549 lung cancer cell line, in which NRF2 is hyperactive (and cannot be further stabilised by NRF2 activators). As shown in Fig. 5B, both CDDO-Me and CDDO-TFEA, but not CDDO, significantly reduce the invasion of A549 cells. Hemin showed a stronger effect than the two CDDO-derivatives, however the difference in the concentrations used (CDDOs 100 nM vs. Hemin 15 μM), makes difficult the comparison. To further confirm the NRF2-independent effect of CDDO-Me and CDDO-TFEA in these models, we analysed the effect of CDDOs on the invasion of A549 NRF2-KO cells, showing similar results. Finally, we repeated the invasion assays in A549 BACH1-KO cells (cell validation in Suppl.S5H). These BACH1-KO cells showed a reduced invasive potential (as expected) and no significant differences in cell invasion were observed between hemin, CDDO-Me, CDDO-TFEA and CDDO treatments, further confirming that the differential effect on cell invasion obtained with the two CDDO-derivatives and CDDO is via BACH1.

## 4 Discussion

Our results demonstrate that CDDO-Me and CDDO-TFEA - but not CDDO - reduce BACH1 nuclear levels, and that this reduction is sufficient for a strong *HMOX1* induction, explaining their greater potency as *HMOX1* inducers in comparison with CDDO. Our data suggest that these two CDDO-derivatives control the nucleocytoplasmic transport of BACH1 either by increasing nuclear export (in a CRM1-independent manner) or by reducing nuclear import. Although we did not identify the exact mechanism(s) involved, we demonstrated that it is different from the one used by hemin and by other described inhibitors, highlighting the need for a better understanding of the pathways controlling BACH1. In that sense, our data show that the basal levels of BACH1 can be regulated by both proteasomal degradation and autophagy, an observation that warrants further investigation. Also, the fact that CDDO-Me and CDDO-TFEA moderately affected total levels of BACH1 in HaCaT cells, but not in the other cell lines tested, suggests that there must be cell line specific BACH1 regulatory mechanisms.

Another possibility is that as CDDOs can activate the ER stress response leading to apoptosis [50], some of the observed effects could be mediated by this pathway. Although the concentration of CDDOs shown to activate the ER stress (1 μM) are 10 times higher than the ones we used (100 nM), we tested whether BACH1 regulation by CDDOs could be an indirect effect of activation of ER stress leading to caspase mediated apoptosis. In Suppl. Fig. S5I we showed that pre-treatment with the pan-caspase inhibitor ZVAD-K does not impair the nuclear reduction and cytoplasmic accumulation of BACH1 in response to CDDO-Me or CDDO-TFEA, further suggesting that ER stress-mediated apoptosis is not involved.

Our results should be taken into consideration in the design of screening strategies to identify compounds that regulate BACH1, as assays that only look at total BACH1 levels could be misleading. As a sidenote, it would be interesting to address whether the accumulation of cytoplasmic BACH1 may have other functions that are unrelated to its well-characterized role as transcriptional regulator.

Another remaining question is why CDDO-Me and CDDO-TFEA inhibit BACH1 while CDDO does not. Based on their similar structures, we propose that the negatively charged carboxyl group of CDDO may interfere with binding to the target protein. In addition, given that CDDO is electrophilic and other electrophilic compounds such as SFN or TBE31 do not affect BACH1, we can conclude that electrophilicity is not sufficient, but whether it is important or necessary is not clear. To test this, we compared the effect of oleanolic acid and its methyl ester, which are structurally very similar to CDDO and CDDO-Me but lack the electrophilic carbon in ring A (structures in Suppl. Fig. S5J), on HMOX1 expression. Both compounds were unable to induce HMOX1 (Suppl. Fig. S5K). These observations suggest that the electrophilic carbon present in CDDO-Me and CDDO-TFEA might be necessary (but not sufficient) to regulate BACH1. This means that within the battery of electrophilic NRF2 activators, it might be possible to find novel dual BACH1/KEAP1 inhibitors.

BACH1 has recently gained visibility as a potential therapeutic target against a variety of conditions ranging from Parkinson's disease [18], bone destructive diseases [24], non-alcoholic steatohepatitis [30], atherosclerosis [31], insulin resistance [28], coronary artery disease [51] and aging related conditions [25]. In addition, BACH1 is a promising therapeutic target against tumour metastasis in various tumour types [40,43,[52], [53], [54], [55], [56]]. Our study demonstrates that both CDDO-TFEA and CDDO-Me are very potent dual KEAP1 and BACH1 inhibitors, and that they reduce lung cancer cell invasion in a BACH1-dependent and NRF2-independent manner. The relevance of BACH1 as a target could explain some of the CDDO-Me and CDDO-TFEA observed therapeutic benefits and provides a rationale for their novel use in other pathologies such as cancer metastasis.

## Funding

This work was supported by the Medical Research Institute of the 10.13039/100008890University of Dundee, 10.13039/501100000289Cancer Research UK (C52419/A22869 and C20953/A18644) (LV and ADK), 10.13039/501100000723Tenovus Scotland (T18/07) (LC) and 10.13039/501100000294Medical Research Scotland (PhD-50058-2019). DO was supported by the 10.13039/501100003554Lundbeck Foundation (R335-2019-2138), 10.13039/100008363Kræftens Bekæmpelse (R279-A16218), the 10.13039/501100009910Brødrene Hartman Fond, the Hørslev Fond, the fabrikant Einer Willumsens mindelegat, and the Eva og Henry Fraenkels Mindefond.

## Authors contribution

LC, RM, KXA, MH, SDN, GN, AEK, EBS, WL, LC and CW conducted the experiments and were responsible for initial data analysis, figure preparation and statistical analysis. CW, VIS, TH and TBP provided resources and technical expertise. LV, DO and ADK had a leading contribution in the design of the study, and an active role in the discussion and interpretation of the whole dataset. LV wrote the original draft of the manuscript. All authors reviewed and edited the manuscript. Funding acquisition LV, DO and ADK. All the authors take full responsibility for the work.

## Declaration of interests

The authors declare the following financial interests/personal relationships which may be considered as potential competing interests: ADK is a member of the Scientific Advisory Board of Evgen Pharma, and a consultant for Aclipse Therapeutics.
