# Supplementary material for: The synthetic triterpenoids CDDO-TFEA and CDDO-Me, but not CDDO, promote nuclear exclusion of BACH1 impairing its activity
Source: Redox Biol. 2022 Mar 17;51:102291. doi: 10.1016/j.redox.2022.102291 (PMC8938334; doi:10.1016/j.redox.2022.102291)
Supplement: Multimedia component 1 [file mmc1.docx]

**Suppl. Figure S1. (A)** HaCaT cells were treated with vehicle (DMSO, 0.1%, v/v) or increasing concentrations of either CDDO, CDDO-Me, CDDO-DFPA or CDDO-TFEA. After five hours cells were lysed and samples were analysed by Western Blot. Upper panel is a representative blot and lower panels show the quantification of BACH1 and NRF2 protein levels normalized for actin levels. Data represent means ± SD (n = 3) and are expressed relative to the DMSO-treated samples. **(B)** HaCaT cells were treated with either DMSO (0.1%, v/v) or different concentrations of CDDO, CDDO-Me, CDDO-DFPA or CDDO-TFEA. After 16 hours cells were harvested and lysed and mRNA levels of *HMOX1* and *AKR1B10* were analysed by real-time qPCR. Data were normalised using *HPRT1* as an internal control (n= 3) and are expressed relative to the DMSO treated sample. *P ≤ 0.05, **P ≤ 0.01, ***P ≤ 0.001, ****P ≤ 0.0001. **(C)** Structures of CDDO, CDDO-Me and CDDO-TFEA. **(D)** HaCaT, H1299 or A549 cells were treated with either DMSO (0.1%, v/v) or different concentrations of CDDO-Me or CDDO-TFEA as indicated. After 48 hours, viability was calculated relative to the DMSO-treated control using Alamar Blue.

**Suppl. Figure S2. (A)**  HaCaT WT or NRF2-KO cells were treated with either DMSO (0.1%, v/v), CDDO-Me (100 nM) *(left panel)* or CDDO-TFEA (100 nM) *(right panel)*. Samples were collected at 8 and 16 hours and mRNA levels of *HMOX1* were analysed via real-time qPCR, using *HPRT1* as an internal control. Data are expressed relative to the DMSO-treated samples in each cell line (t=0), which was set to 1.  **(B)** Left panel: Control (WT) and NRF2 gain-of-function (GOF) HK2 cells were treated with either DMSO (0.1%, v/v) or sulforaphane (SFN). Three hours later the levels of NRF2 were measured by western blot. Right panel: Basal mRNA levels of *HMOX1* and *AKR1B10* in control (WT) and NRF2-GOF HK2 cells were analysed by RT-qPCR. *HPRT1* was used as a housekeeping gene for the analysis. ***P ≤ 0.001. **(C)** HK2 Control (WT) and NRF2-GOF cells were treated with DMSO, CDDO (100 nM), CDDO-Me (100 nM), CDDO-TFEA (100 nM) or Hemin (10 μM) for 16h. *AKR1B10* mRNA levels were analysed using RT-qPCR and *HPRT1* as a housekeeping gene. Data are expressed relative to the DMSO-treated samples in each cell line (DMSO in WT and NRF2-GOF cells set to 1).

**Suppl. Figure S3. (A)** HaCaT WT cells were treated with either DMSO (0.1%, v/v), CDDO (100 nM), TBE-31 (100 nM) or CDDO-TFEA (100 nM) for six hours. Nuclear and cytosolic fractions were isolated and analysed for their levels of BACH1. Left panel is a representative blot and right panels show the quantification of BACH1 nuclear and cytoplasmic levels (n=2), normalized against their respective loading controls. Data is expressed relative to the DMSO treated samples.

**Suppl. Figure S4. (A)** HaCaT cells were incubated with either DMSO (0.1%, v/v), MG132 (10 μM) or MLN4924 (2 μM) for one hour. After that, either DMSO (-), CDDO-Me (100 nM) or CDDO-TFEA (100 nM) was added. Six hours later, cells were harvested and analysed for their levels of BACH1 and NRF2. Left panel is a representative blot and right panel is the quantifications of total BACH1 levels normalised against ACTIN. Data represent means ± SD (n= 3) and are expressed relative to the DMSO sample. **(B)** HaCaT cells were incubated with either DMSO (0.1%, v/v) or bafilomycin A1 (BAF-A1, 100 nM). Two hours later they were treated with either DMSO (-), CDDO-Me (100 nM) or CDDO-TFEA (100 nM) for another six hours. Subcellular fractionation was performed as previously described. Upper panel is a representative blot and lower panels are the quantifications of nuclear and cytoplasmic BACH1 levels normalised against their corresponding loading control. Data represent means ± SD (n= 2) and are expressed relative to the DMSO sample. Control and BAF-A1 treated samples were all loaded in the same gel. **(C)** As in (B) but whole cell extract was analysed **(D)** HaCaT cells were treated with either DMSO (0.1%, v/v), CDDO (100 nM), CDDO-Me (100 nM) or CDDO-TFEA (100 nM) for 3h or 16h. Samples were collected and mRNA levels of BACH1 were analysed via real-time qPCR, using HPRT1 as an internal control. Data are expressed relative to the DMSO-treated samples at each time point. **(E)** HaCaT cells were incubated with either DMSO (0.1%, v/v), cycloheximide (CHX, 10 μM) for 2h or actinomycin D (ActD, 1 μg/mL) for 30 min. After that, either DMSO (-), CDDO-Me (100 nM) or CDDO-TFEA (100 nM) was added. Six hours later, cells were harvested and nuclear/cytoplasmic fractions were isolated and analysed for their levels of BACH1 and NRF2. Upper panel is a representative blot; lower panels are the quantifications of nuclear and cytoplasmic BACH1 levels normalised against their corresponding loading control. Data represent means ± SD (n= 3) and are expressed relative to the DMSO-treated cells. **(F)** HaCaT cells were incubated with either DMSO (0.1%, v/v), leptomycin B (Lepto, 25 ng/mL) or KPT-330 (1 μM). After two hours, either DMSO (-), CDDO-Me (100 nM) or CDDO-TFEA (100 nM) was added. Six hours later cells were harvested and subcellular fractionation was performed. BACH1 and NRF2 protein levels were analysed by western blot. Upper panel is a representative blot and lower panels are the quantification of BACH1 nuclear and cytoplasmic levels normalised to the corresponding loading control. Data represent means ± SD (n=3) and are expressed relative to the DMSO-treated cells. **(G)** HaCaT cells were incubated with either DMSO (0.1%, v/v), leptomycin B (Lepto, 25 ng/mL) or KPT-330 (1 μM). After two hours, either DMSO (-), CDDO-Me (100 nM) or CDDO-TFEA (100 nM) was added. Six hours later cells were harvested and BACH1 and NRF2 protein levels were analysed by western blot. Left panel is a representative blot and right panel is the quantification of total BACH1 levels normalised to ACTIN. Data represent means ± SD (n=3) and are expressed relative to the DMSO-treated cells.

**Suppl. Figure S5. (A)** HK2 cells were treated with DMSO, CDDO (100 nM), CDDO-Me (100 nM), CDDO-TFEA (100 nM) or hemin (10 μM) for 6h. Cells were lysed and total BACH1, NRF2 and actin levels were analysed by western blot. Representative blot is shown on the left panel and quantification of BACH1 protein levels (n=2) is in the middle panel. Right panel: HK2 cells were treated with either DMSO (0.1%, v/v), CDDO (100 nM), CDDO-Me (100 nM), CDDO-TFEA (100 nM) or hemin (10 μM) for 16h. *HMOX1* mRNA levels were analysed. *P ≤ 0.05, **P ≤ 0.01. **(B)** A549 cells were treated with DMSO (0.1%, v/v) or increasing concentrations of CDDO-Me or CDDO-TFEA for 6h. Left panel shows a representative blot while middle panels show quantification of BACH1 protein levels (n= 2). Right panel: A549 cells were treated with DMSO (0.1%, v/v), CDDO (100 nM), CDDO-Me (100 nM) or CDDO-TFEA (100 nM) for 6h. *HMOX1* mRNA levels were analysed. ***P ≤ 0.001 **(C)** H1299 cells were treated with DMSO (0.1%, v/v) or increasing concentrations of CDDO-Me or CDDO-TFEA for 16h. Left panel shows a representative blot while middle panels show quantification of BACH1 protein levels (n=2). Right panel: H1299 were treated with DMSO (0.1%, v/v), CDDO (100 nM), CDDO-Me (100 nM) or CDDO-TFEA (100 nM) for 16h. *HMOX1* mRNA levels were analysed. **P ≤ 0.01, ***P ≤ 0.001. **(D)** LX2 cells were treated with DMSO (0.1%, v/v), CDDO (50 nM), CDDO-Me (50 nM), CDDO-DFPA (50 nM) or CDDO-TFEA (50 nM) for 6h. Samples were lysed and total BACH1, NRF2 and ACTIN levels were analysed by western blot. Representative blot is shown on the left panel and quantification of BACH1 protein levels (n= 3) is in the middle panel. Right panel: LX2 cells were treated with either DMSO (0.1%, v/v), CDDO (50 nM) or CDDO-TFEA (50 nM) for 16h. *HMOX1* mRNA levels were analysed. *P ≤ 0.05, **P ≤ 0.01. **(E)** HK2 cells were treated with DMSO (0.1%, v/v), CDDO-Me (100 nM) or CDDO-TFEA (100 nM). Six hours later cells were harvested and subcellular fractionation was performed. BACH1 protein levels were analysed via western blot. Panels on the left show a representative blot; panels on the right are the corresponding BACH1 nuclear and cytoplasmic quantifications, which were normalised against their internal control (i.e., LAMIN for nuclear and TUBULIN for cytoplasmic levels). Data represent means ± SD (n= 3) and are expressed relative to the DMSO-treated samples**. (F)** LX2 cells were treated with DMSO (0.1%, v/v), CDDO-Me (50 nM), CDDO-DFPA (50 nM) or CDDO-TFEA (50 nM). Six hours later cells were harvested and subcellular fractionation was performed. BACH1 protein levels were analysed via western blot. Panels on the left show a representative blot; panels on the right are the corresponding BACH1 nuclear and cytoplasmic quantifications, which were normalised against their internal control (i.e., LAMIN for nuclear and TUBULIN for cytoplasmic levels). Data represent means ± SD (n= 3) and are expressed relative to the DMSO-treated samples. **(G)** H1299 cells were treated with DMSO (0.1%, v/v), CDDO-Me (100 nM) or CDDO-TFEA (100 nM). Six hours later cells were harvested and subcellular fractionation was performed. BACH1 protein levels were analysed via western blot. Panels on the left show a representative blot; panels on the right are the corresponding BACH1 nuclear and cytoplasmic quantifications, which were normalised against their internal control (i.e., LAMIN for nuclear and TUBULIN for cytoplasmic levels). Data represent means ± SD (n= 3) and are expressed relative to the DMSO-treated samples. **(H)** BACH1 protein levels in A549-WT and A549 BACH1 KO were analysed via western blot. Total cell lysates are shown.

**(I)** HaCaT cells were incubated with either DMSO (0.1%, v/v) or Z-VAD(OMe)-FMK (20 μM). After one hour, either DMSO (-), CDDO-Me (100 nM) or CDDO-TFEA (100 nM) was added. Six hours later cells were harvested, and subcellular fractionation was performed. BACH1 and NRF2 protein levels were analysed by western blot. Upper panel is a representative blot and lower panels are the quantification of BACH1 nuclear and cytoplasmic levels normalised to the corresponding loading control. Data represent means ± SD (n=3) and are expressed relative to the DMSO-treated cells. **(J)** Structures of Oleanolic acid and oleanolic acid methyl ester. **(K)** HaCaT cells were treated with either DMSO (0.1%, v/v), oleanolic acid or oleanolic acid-Me at the indicated concentrations for 8h. mRNA levels of *HMOX1* were analysed by qRT-PCR, using *HPRT1* as a housekeeping gene.
